# An Overview of the Genetic Structure within the Italian Population from Genome-Wide Data

**DOI:** 10.1371/journal.pone.0043759

**Published:** 2012-09-12

**Authors:** Cornelia Di Gaetano, Floriana Voglino, Simonetta Guarrera, Giovanni Fiorito, Fabio Rosa, Anna Maria Di Blasio, Paola Manzini, Irma Dianzani, Marta Betti, Daniele Cusi, Francesca Frau, Cristina Barlassina, Dario Mirabelli, Corrado Magnani, Nicola Glorioso, Stefano Bonassi, Alberto Piazza, Giuseppe Matullo

**Affiliations:** 1 Department of Genetics, Biology and Biochemistry, University of Turin, Turin, Italy; 2 Human Genetics Foundation (HuGeF), Turin, Italy; 3 Istituto Auxologico Italiano, Cusano Milanino, Italy; 4 Banca del Sangue, San Giovanni-Molinette Hospital, Turin, Italy; 5 Department of Health Sciences, University of Eastern Piedmont, Novara, Italy; 6 Department of Medicine, Surgery and Dentistry, University of Milan, Milan, Italy; 7 Genomic and Bioinformatics Unit, Fondazione Filarete, Milan, Italy; 8 Unit of Cancer Epidemiology, University of Turin and CPO-Piemonte, Turin, Italy; 9 Department of Translational Medicine, University of Eastern Piedmont, Novara, Italy; 10 Hypertension and Related Diseases Center, AOU, University of Sassari, Sassari, Italy; 11 Unit of Clinical and Molecular Epidemiology, IRCCS San Raffaele Pisana, Rome, Italy; University of Florence, Italy

## Abstract

In spite of the common belief of Europe as reasonably homogeneous at genetic level, advances in high-throughput genotyping technology have resolved several gradients which define different geographical areas with good precision. When Northern and Southern European groups were considered separately, there were clear genetic distinctions. Intra-country genetic differences were also evident, especially in Finland and, to a lesser extent, within other European populations. Here, we present the first analysis using the 125,799 genome-wide Single Nucleotide Polymorphisms (SNPs) data of 1,014 Italians with wide geographical coverage. We showed by using Principal Component analysis and model-based individual ancestry analysis, that the current population of Sardinia can be clearly differentiated genetically from mainland Italy and Sicily, and that a certain degree of genetic differentiation is detectable within the current Italian peninsula population. Pair-wise F_ST_ statistics Northern and Southern Italy amounts approximately to 0.001 between, and around 0.002 between Northern Italy and Utah residents with Northern and Western European ancestry (CEU). The Italian population also revealed a fine genetic substructure underscoring by the genomic inflation (Sardinia vs. Northern Italy = 3.040 and Northern Italy vs. CEU = 1.427), warning against confounding effects of hidden relatedness and population substructure in association studies.

## Introduction

Genetic gradients are represented by continuous differences in allele frequencies created by events such as gene flow between two different populations, or by a demographic expansion into a scarcely populated environment, leading to a partial admixture with indigenous populations, genetic drift or differential selection [1]. These differences in allele frequencies may generate population stratification, which is an important confounding factor in genetic association studies [2]. The genetic composition of contemporary Europeans has been repeatedly studied using particular sets of markers that recent technologies have unveiled. Genetic differences between populations have been investigated by Menozzi *et al.* using gene frequencies of 38 classical pre-molecular markers [3] and by Ammerman *et al.* who described that the genetic composition of contemporary Europeans may have been shaped by a prehistoric demic diffusion that drove the expansion of agriculture [4].

Several studies have been carried out in the past 20 years focused on Y-chromosomal haplogroups and mtDNA across Europe [5], [6], [7], [8], [9], [10], [11], [12], [13], [14]. Y chromosome markers are more geographically clustered, while the pattern of variability of mtDNA seems to be less spatially structured, although these single locus markers are easily subjected to genetic drift. Advances in high-throughput genotyping technology have provided greater information on differences between populations, and at the same time have shown that genetic gradients exist and correspond well to geographical areas [15], [16], [17], [18], [19], [20]. However, genetically homogeneous populations do not always coincide with the ‘political’ definition of a country, but a recent paper has shown that clinal patterns in principal component analysis (PC) probably develop due to a simple isolation-by-distance process [21].

An initial overall representation of the European population structure on a fine-spatial scale was demonstrated by [15]. In this paper the first principal component (PC1) axis aligns with the North-Northwest/South-Southeast direction, possibly justified by a special role for this geographic axis in the demographic history of Europeans. Finland was found to be the European population within single country genetic differences [20], [22], [23], [24], [25]. Differences between regions of the same country have also been shown within the population of the British Isles [26], [27], [28], as well as in the Swedish population [29], [30], in Estonia [16] and Iceland [31]. Using genome-wide SNPs data, Nelis and colleagues have also shown a population structure on a fine–spatial scale in Italy, with a remarkable distinction between Southern Italians and other European populations [16]. In this scenario, using classical genetic markers [32], [33], [34], there is a certain degree of genetic substructure within Italy, especially between Sardinia and the Italian mainland.

Considering single nucleotide polymorphisms (SNPs) located in the non-recombining region of the Y chromosome (NRY), Italy remains within the range of European Y-chromosome variability, although a non-random distribution of Y markers was observed with more than 70% of Y chromosome diversity distributed along the North-South axis of the Italian peninsula [35]. One Y chromosome lineage, I-M26, is very common (40.9% of the population) in Sardinia [5], and it is also detected in some Western European populations, but with lower frequencies [36], like in the Bearnais (7.7%) or in the Basque, (Spanish and French) (6.0%) populations [6], [36], [37], [38]. The distribution of this lineage in Europe indicates that M26 mutation occurred in a I1b Y chromosome from Western Europe, most likely in a population in Iberia/Southern France before the main initial peopling of Sardinia [39].

Geographical patterns of mtDNA variations within the Italian peninsula showed North-South clines with clear differences between Sardinian and the mainland populations [40].

Using genome-wide SNPs data, Nelis and colleagues have also shown a population structure on a fine–spatial scale in Italy, with a remarkable distinction between Southern Italians and other European populations [16].

We have investigated the genetic structure of the Italian population on a finer scale with respect to previous work by utilizing a greater number of markers and including more individuals in the study, to the best of our knowledge this is the first genome-wide SNP-based study focusing specifically on Italy. We estimate genetic differentiation among Italian samples and between Italian and other populations from the literature: 1) using a model free analysis like PC and a model-based analysis to infer individual ancestry components (ADMIXTURE software), 2) by calculating pair-wise F_ST_ statistics and estimating the identity-by-state (IBS) sharing between and within populations, 3) by estimating the genomic inflation factor in order to assess the effect of population stratification.

## Materials and Methods

### Study samples and data sets

DNA samples were obtained from 49 unrelated volunteers from four different Italian macro-areas (Tuscany, Sicily, Piedmont and Sardinia). Details of the affiliation of the municipalities within the macro-areas mentioned in this work are described in Figure S2.These individuals were grouped according to their birth place, and were selected to have their parents and four grandparents born in the same region. This small sample set is not a random sample of the modern, admixed population, but rather it should approach the historical population structure.

In addition we used existing genetic data relative to control subjects from both published and unpublished genome-wide association studies: a study on malignant pleural mesothelioma (MESO Study, [41]; a study on obesity (GEO-IT, Di Blasio at Auxologic Institute in Milan, unpublished); a study on hypertension (HYPERGENES [42]). For these control samples, we retrieved information on the birth place, thus possibly also including children of first generation migrants who were born in a different place from their parents. All participants signed an informed consent in agreement with the guidelines of the ethical committees of the institutions involved. An internal ethical review board at HuGeF foundation (Comitato Etico HUGEF/15-12-2011) approved the study. An ethical revision of the internal ethical steering board of the HYPERGENES STUDY, GEO-IT study and MESO study approved the entire process for each dataset. The complete dataset after validation was of 1,014 Italian samples.

We also included genotypic data from other populations freely available from the literature and living in the Mediterranean basin, like 57 CEU individuals from the HapMap project (Phase 2; release 23) [43], (Table 1); In addition, 134 individuals coming from the Middle East (Bedouin from Negev, Israel; Druze from Carmel, Israel; Palestinian from Israel) were included; 29 volunteers from Northern Africa (Mozabite from Mzab, Algeria) and 28 subjects from France from the Human Genome Diversity Panel (HGDP-CEPH) [44], [45]. Samples from the literature were included in this study to increase the sample size for Italy, in order to compare Italy with other European populations and the Mediterranean basin and finally to estimate the degree of stratification between the CEU sample and Italy. In Table 1 a list of samples and data sets analyzed in our study is reported.

**Table 1:** Datasets and individuals number from each population.

	N-IT	C-IT	S-IT	SAR	CEU	FRE	PAL	BED	DRU	MOZ
**HapMap**		76			57					
**HGDP**	13	6		28		28	46	46	42	29
**MESO**	277	19	58	7						
**Our sample**	11	22	13	3						
**GEO-IT**	221	9	21	5						
**HYPERGENES**				225						
**tot**	522	130	92	268	57	28	46	46	42	29

Individuals included were filtered for individual call rate >98% and Identity By State (IBS) >0.05. Individuals included belong to Northern Italian (N-IT), Central Italian (C-IT), Southern Italian (S-IT), Sardinian (SAR), CEU HapMap (CEU), French (FRE), Palestinian (PAL), Bedouin (BED), Druze (DRU), and Mozabite (MOZ).

### DNA extraction and genotyping

DNA was purified from blood samples by a standard phenol/chloroform extraction method. DNA concentrations were determined by spectrometry (NanoDrop 8000, Thermoscientific). The Illumina HumanOmni 1–QUAD, v1.0 BeadChip Array (Illumina Inc, S. Diego, CA, USA) was used to genotype 1,140,419 SNPs on 49 unrelated volunteers. Genotyping, according to the instructions provided by the manufacturer, was carried out at the Human Genetics Foundation (HuGeF) in Turin. All the information about this data set is reported in Table S1.

### Quality Assessment and Control Procedure

Array-based SNP genotypes were subjected to stringent quality control, performed separately for each panel (Table S1). Samples that were too closely related to another sample (identity-by-state distance IBS <0.05) were removed. We also removed samples with genotype missing data >2%. Furthermore, we removed SNPs that had a minor allele frequency (MAF) <0.01 in all combined studies, or that failed Hardy-Weinberg equilibrium (HWE) with P< = 0.001. The average proportion of heterozygous genotypes at X chromosome, as described by Lao et al. 2008 [20], was used to avoid false gender assignments. Data management and quality control were carried out using the PLINK toolset [46]. For the present study we used only autosomal SNPs. The intersection between different data sets was of 163,355 SNPs and after three other steps MAF, HWE test, and *linkage disequilibrium* (LD) pruning, we used 125,799 SNPs in: 1,262 individuals in the European and Mediterranean dataset, 1,099 in the European data set, and 1,014 in the Italian dataset (746 in the Italian dataset excluding Sardinia).

### Statistical Data Analyses

#### Principal Component Analysis

Principal Component Analysis was performed on a set of about 125,799 pruned markers. The pruning procedure was used to optimize the analysis of population structure, identifying a set of SNPs with low background LD (r^2^ = 0.5).

To perform the analysis we used the function *prcomp* of R (package *mva*) [47], where the calculation is carried out by a singular value decomposition of the (centered and scaled) data matrix. We further confirmed PCA clustering by the K-means analysis [48]. The K-means clustering was calculated using the R package stats.

#### IBS analysis

The identity-by-state (IBS) sharing analysis [49] at both individual and population levels was performed. By using PLINK [46], the genome-wide average proportion of alleles sharing IBS was calculated for all subject pairs among the combined data sets. We further characterized the empirical distributions of IBS sharing within and between populations by using density estimation using the function of R (package *stats*) [47].

### ADMIXTURE

The software ADMIXTURE [50] implements a model-based clustering method for estimating ancestry using autosomal genotype data consisting of unlinked markers. We included 126K SNPs resulting after the LD pruning. The analysis uses a cross-validation procedure to validate results, it was run with the number of clusters, K, from 2 to 10; we chose as termination criteria when the log-likelihood change between interactions falls below 0.001 and converged after 100 interactions.

### F_ST_ and Mantel test

To estimate F_ST_ pairwise values between populations the Eigensoft program (Patterson et al, 2006) was used. High F_ST_ values implie a high degree of genetic differentiation among populations.

The Mantel test was calculated by using the R package *adegenet* [51]. It was used to test the relationship between the first two PC scores and the latitude and longitude. Finally, we computed the correlation between genetic distance (measured through Identity by Status matrix of PLINK), and geographical distance matrix of individuals (calculated with *dist* function of the R software, taking into account latitude and longitude of birth place).

### Genomic control

We calculated the maximum possible inflation factor [52] between pairs of populations using PLINK [46].

## Results

### Principal component analysis of the Italian population

**Figure 1:**
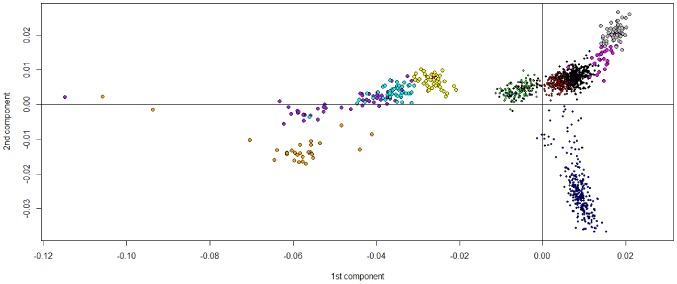
SNP-Based PC of 1,262 individuals from 10 sub-populations. The Italian population plotted onto the first two principal components defined by the European HGDP-CEPH populations and CEU HapMap data. Scatter plot of the first two principal components, obtained using R software (*prcomp*). Analysis based on 125,799 autosomal SNPs. Individuals included belong to Northern Italy (N-IT): black dots, Central Italy (C-IT): red dots, Southern Italy (S-IT): green dots, Sardinian (SAR): blue dots, CEU HapMap (CEU); light blue dots, Beduoin (BED): purple dots, Druze (DRU): yellow dots, Mozabite (MOZ): black triangles, Palestinian (PAL): red triangles, French (FRE): green triangles. The top 100 Eigenvectors and associated Eigenvalues for this plot are given in Supplementary Material: Figure S2 (panel a).

The eigenvectors for different subsets (HGDP-CEPH data, HapMap CEU and Tuscany data) were calculated in order to project the Italian data sets onto a two dimensional space (Figure 1) using 125,799 autosomal SNPs. The top 100 PCs were generated using the R software; we however focused on the top five, since the eigenvector values remained relatively constant in subsequent PCs, as indicated by the screeplot (Figure S1: top panel a); European dataset).

The position of the Italian population samples suggests that genetic distances between these populations and other European and Middle East populations has a good correlation with geographic distances. At the same time, Sardinia was confirmed to be a genetic “outlier”.

**Figure 2:**
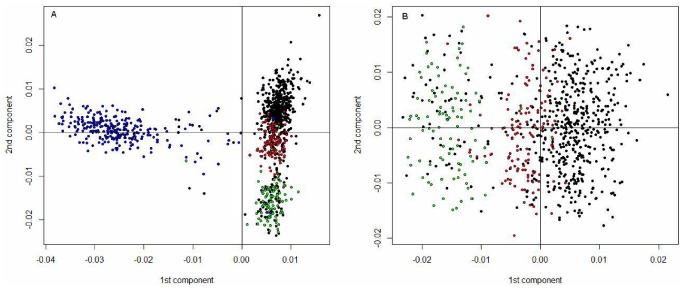
SNP-Based PC of 1,014 individuals from the Italian dataset. A. A Scatter Plot of the Italian population of the first two principal components obtained via R software (*prcomp*). Individuals included belong to Northern Italy : black dots, Central Italy : red dots, Southern Italy : green dots, Sardinian: blue dots. B. Italian population without the Sardinian-projected scatter plot of the first two principal components obtained via the R software (*prcomp*). Both analyses were based on 125,799 autosomal SNPs and 1,014 individuals for the Italian dataset and 746 individuals for the Italian dataset without Sardinia. Top 100 Eigenvectors and associated Eigenvalues for this plot are given in Supplementary Material: Figure S2 (panel b and c, respectively).

Our main goal was to investigate the genetic structure of the Italian population considering four main macro-areas (Northern, Central, Southern Italy and Sardinia). We carried out PC analysis on the Italian samples and plotted the eigenvectors 1 and 2 in Figure 2. Most samples fell within a main cluster which seems to be indicative of Italian peninsula individuals. The first PC divided Italian populations in two clusters, one for Sardinia and the other for the remaining three Italian macro-areas. The Sardinian population is highly dispersed along the first eigenvector.

The second PC divided Italian mainland population into two clusters, with a certain degree of overlapping between Northern and Central Italy, and a separate cluster for Southern Italy, suggesting that genetic variation is generally continuous rather than discrete, at least within Italian mainland. In order to quantify the effect of migration we have done a correlation test [53] between PC1 or PC2 scores and geographical distance, Table S2 (Mantel test 1000 permutations PC1 R = 0.32, p-value = 2.2_*_e^−16^ and PC2 R = 0.49 p-value = 2.2_*_e^−16^). Mantel test was also used to compare genetic distance identities by state (IBS) between individuals and geographic distance: it showed a good correlation with geographic distance between sampled individuals (R = 0.41, p-value = 2.2_*_e^−16^, Table S2). In the same table we also showed a correlation between PC1 and PC2 scores with latitude and longitude.

Although a correlation between PCs and geography have been observed within the country (Figure 2), it is difficult, at this stage, to improve the accuracy at a regional level and perhaps a better clustering could be achieved by increasing the number of samples for each region (Figure S2).

The individuals' geographical clustering is mostly attributable to PC1 and PC2 while the subsequent components are influenced by particular regions along the genome. To further confirm this assumption, we calculated PC3 and PC4 on the Italian dataset and noted that they were also not stratified by a population label and at the same time we also observed that by using the K-means, it is possible to differentiate 3 clusters on PC4 (Figure S3). We have then plotted the contribution of each SNP for the first four PCs, against the genomic location, making a Manhattan plot (Figure S4).We showed that for PC3 and PC4, the top SNPs localize to chromosome 8 between 8.135 and 11.90 Mb.

This genomic region is characterized by a large inversion with an unusual linkage disequilibrium (LD) pattern. Considering the state of this inversion, three different orientations of this region of DNA can be observed, i.e. inverted homozygous, heterozygous, or homozygous non-inverted. Using only the SNPs within this region (163 SNPs),those that contribute mainly to the PC4, we reiterated the PC Analysis (Figure S5 panel A). We chose 6 HapMap CEU individuals predicted to be homozygous inverted, heterozygous, or homozygous non-inverted on chromosome 8p23.1 from the literature [54].

Using these individuals as a reference we roughly estimated the frequency of these genotypes by the K-means clustering (Figure S5 panel B). The estimated frequency of homozygous-inverted was 21%; 31.2% for homozygous non-inverted; and 47.9% for heterozygous individuals, on the entire Italian dataset. However, it must be emphasized that the PC analysis does not calculate frequency, rather it shows stratification of the genotype inversions. The real percentage of the three genotypes can only be obtained experimentally, as in Deng et al. 2008 [55] and more recently in Salm et al.2012 [56].

### Model-based ancestry analysis

**Figure 3:**
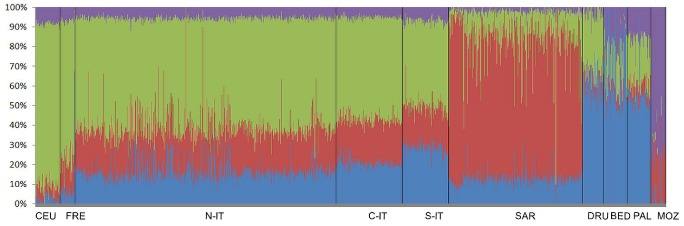
Identity-by-state (IBS) sharing between and within populations. Density estimates for empirical distributions of genome-wide mean proportions of alleles sharing identity-by-state between subjects from different population or within the same populations, are shown for A) Northern Europe (CEU and French), B) Middle East (Bedouin, Palestinian and Druze), C) Northern Africa (Mozambite), D) within Italian populations. Color code as in Figures 1 and 2.

We used the ADMIXTURE software [50] calculating the ancestry fractions for each individual included in the analysis (Fig. 3). This software returns a cross-validation error value for each number of ancestral populations assumed for the analysis. The number of markers needed to resolve populations is generally inversely proportional to the genetic distance between the populations. By using 126K autosomal SNPs, we obtained at K = 4 the lowest cross-validation error. The HapMap CEU individuals showed an average Northern Europe (NE) ancestry (light green) of 83%. A similar pattern is observed in French, Northern Italian and Central Italian populations with a NE ancestry of 70%, 56% and 52% respectively (Figure 3). According to the PCA plot, also in the ADMIXTURE analysis there are relatively small differences in ancestry between Northern Italians and Central Italians while Southern Italians showed a lower average admixture NE proportion (43,6%) than Northern and Central Italy, and a higher Middle East ancestry (light blue) of 28%. The Sardinian samples display a pattern of crimson common to the others European populations but at a higher frequency (70.4%). The HGDP-CEPH Bedouins population showed a strong population substructure and apparently consisted of two different subpopulations on the basis of the percentage of the Middle East ancestry (light blue) and NE ancestry (light green). One of the clans was more similar to Palestinians. The HGDP-CEPH Mozabite population have an admixture proportion from Northern Africa (purple) of 73.2%. Figure S6 shows the results from K = 2 to K = 9. A plot of the distribution of cross-validation error estimate is shown in Figure S7.

### IBS analysis

**Figure 4:**
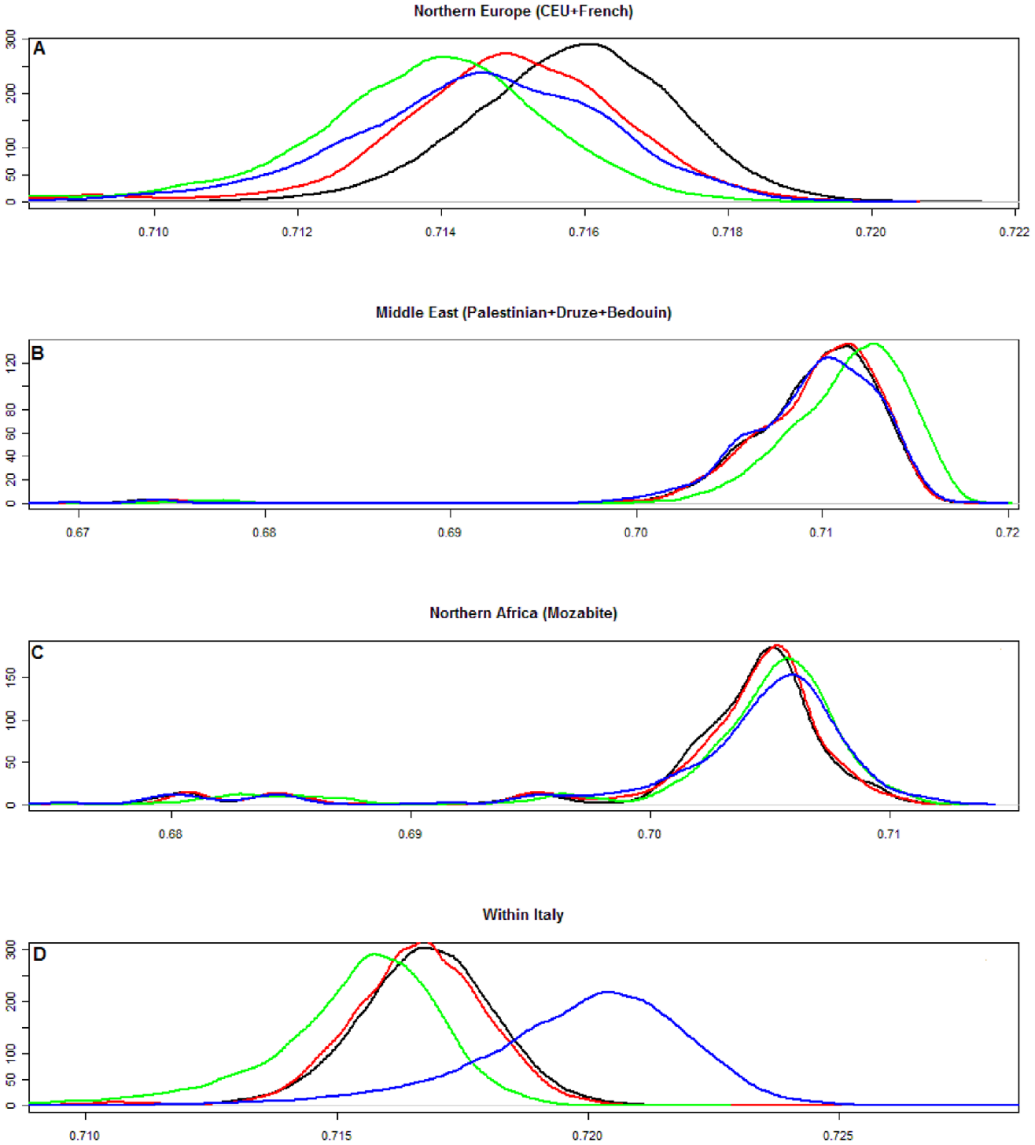
Clustering of the European, Northern African and Middle Eastern individuals by the Structure software. Model-based ancestry analysis based on a subset of HGDP-CEPH and HapMap CEU data using the merged data of 126K autosomal SNPs. Ancestry for each individual was inferred using ADMIXTURE [50] at K = 4. Abbreviations as in Figure 1.

Distributions of IBS sharing between and within population are shown in Figure 4 for Northern Europe (CEU HapMap and French from HGDP-CEPH), for Middle Eastern populations, for Northern Africa populations (Mozabite) and Northern, Central Southern Italy and Sardinia. This analysis used data from 125,799 autosomal SNPs.

Density estimates for empirical distributions of genome-wide mean proportions of alleles shared between Italian and Northern European populations (Figure 4, top panel a) show that the median IBS sharing was higher for Northern Italy and lower for Sardinia and Southern Italy. Conversely, the median IBS sharing between Middle Eastern populations was higher for Southern Italy than for Northern Italy (Figure 4, center panel b). It was also possible to appreciate a lower IBS sharing between Mozabite populations (Figure 4, panel c).

In the Figure 4 bottom panel (D) the mean IBS sharing between pairs of individuals within each Italian population was described: the mean IBS was highest for Sardinians (0.72 with a standard error 10_*_e^−6^, range 0.706–0.729), followed by Northern and Central Italy (both 0.717 with a standard error 4_*_e^−6^ and 4_*_e^−5^ and a range of 0.710–0.723 and 0.708–0.723, respectively) and then Southern Italy (0.715 with a standard error 2.6_*_e^−5^ a range of 0.707–0.722). The distribution mode was similar and higher for the populations of the Italian Peninsula, and lower for the Sardinians.

These results are consistent with the model-based ancestry analysis and with the position of these populations in the eigenvector PC space.

### F_st_ analysis

We quantified genetic differentiation between the Italian population and the subset of HGDP-CEPH populations from Northern Europe (French), the Middle East (Druze, Palestinians and Bedouin), Northern Africa (Mozabite,) and from HapMap CEU, by calculating the pair-wise F_ST_ statistics. Estimates are given in Table 2. Notably, the genetic distance between Sardinia and each of the mainland Italian populations (F_ST_ = 0.004) was slightly lower than for many other European population pairs separated by larger geographical distances, for example, Southern Italians and CEU (F_ST_ = 0.005). In general, F_ST_ was lower between population pairs in closer geographical proximity like Southern Italians vs Central Italians (F_ST_ = 0.001). Southern Italians showed a genetic affinity with Middle East populations, such as Palestinian and Druze; and Northern Italian populations were genetically closer to the French and CEU populations. However, it should be stressed that the estimates are less accurate for pairs involving a population with a small sample size.

**Table 2:** F_st_ values and genomic control inflation factor (λ_GC_) between National areas.

	N-IT	C-IT	S-IT	SAR	CEU	BED	DRU	MOZ	PAL	FRE
**N-IT**	0	0	0.001	0.004	0.002	0.015	0.011	NA	0.01	0.001
**C-IT**	1.120	0	0.001	0.004	0.004	0.014	0.01	NA	0.009	0.002
**S-IT**	1.247	1.113	0	0.004	0.005	0.011	0.008	NA	0.006	0.003
**SAR**	3.040	2.213	1.878	0	0.009	0.018	0.014	NA	0.013	0.006
**CEU**	1.427	1.517	1.676	2.562	0	0.022	0.017	NA	0.016	0
**BED**	3.329	2.831	2.169	3.689	3.177	0	0.013	NA	0.008	0.019
**DRU**	2.578	2.220	1.790	2.985	2.654	2.073	0	NA	0.009	0.014
**MOZ**	3.961	3.569	2.980	**4.037**	3.611	2.508	3.019	0	NA	NA
**PAL**	2.574	2.174	1.703	3.028	2.663	1.677	1.831	2.529	0	0.014
**FRE**	**1.102**	1.153	1.293	1.590	1.044	2.308	1.998	2.818	1.968	0

F_st_ values above the diagonal; λ**_GC_** below. Individuals included belong to Northern Italian (N-IT), Central Italian (C-IT), Southern Italian (S-IT), Sardinian (SAR), CEU HapMap (CEU), Bedouin (BED), Druze (DRU), Mozabite (MOZ), Palestinian (PAL), and French (FRE) populations.

### Population stratification analysis

Population stratification refers to a situation in which subgroups of individuals within the population of interest are, on average, more closely related to each other than to other individuals of the wider population. These allele frequency differences, can bias testing results and lead to artifact associations in case control studies. The genomic control inflation factor (λ**_GC_**) was calculated to evaluate the possible impact of population stratification inside the four defined Italian subpopulations, and other populations from the literature. Results are shown in Table 2. The highest lambda value within Italy was 3.040, between Sardinia and Northern Italy. Moreover, other combinations also show substantial inflation, such as Southern vs Northern Italy (λ_GC_ = 1.247), and warns against the confounding effects of hidden relatedness and population substructure in association studies. As far as European and Middle Eastern or North African populations are concerned, results showed that a random Northern Italian population was well matched to the French population (λ_GC_ = 1.102), but this does not correspond to the comparison between Northern Italians and CEU from HapMap (λ_GC_ = 1.427).

## Discussion

In this study, a genome-wide analysis of population structure within the Italian population from autosomal SNP data is presented for the first time. Using data from Human Genome Diversity Panel (HGDP-CEPH) [44], [45], [57] and from the HapMap Projects [43], we performed an ancestry analysis and PC analysis (Figure 1). We projected the Italian population onto a “map” defined by the first two PC, based on the European subset of the HGDP-CEPH dataset and some HapMap populations. The relative position of the samples reflected their geographic location: the close correlation between PC and geography, was previously reported by several authors [15], [16], [20], [27], [58], [59]. When compared to other European populations, Sardinia was confirmed to be a genetic “outlier”, whereas the Northern Italian population was genetically close to the French population, and the Southern Italians had some similarities with other Mediterranean populations such as those from Middle East. Unfortunately, lack of data from other relevant reference populations from the South-East Europe, e.g. from the Balkan peninsula, made it impossible to fully analyze the extent of the Eastern contribution in Italian populations. We also only made the PC analysis on Italian datasets with and without Sardinia (Figure 2). The first two PCs identified a good correlation with geographical distance and discriminate at least three of the four macro-areas within the Italian peninsula: Northern and Central, Sardinia and Southern Italy. Both analyses (Figure 1 and Figure 2, panel a) confirm the differentiation of Sardinia. In the PC analysis there is an appreciable degree of overlap between individuals born in Northern Italy but with a Southern ancestry (Figure 2, panel a), which could be explained by internal migration occurred during the last two generations, where people from Southern Italy have left their place of origin to look for better economic opportunities in other Italian regions.

A finer view of the Italian substructure, can be seen in Figure S2 where the hidden population structure within the Italian dataset is appreciable. Subjects are labeled by municipality, or in the case of the Sardinian samples, by the main linguistic area. In this figure we can appreciate the lack of clustering at the municipality level, also within Sardinia. Individuals seem to cluster within the main macro-area, but the geographic patterning is less obvious for the municipality (or in the case of Sardinia, linguistic) division, and in our opinion this pattern indicates no substructure within regions among municipalities, while the structuring between regions can be easily detected. It is also possible appreciate a certain genetic homogeneity within Sardinia.

The genetic structure observed in our dataset is expected to be mainly a consequence of demographic processes such as internal migration within and between the macro-areas. Indeed, Italy remains characterized by a strong migratory movements of the population within its territory [60] that was particularly significant from 1959–1970, but still present nowadays. Some authors [15] have calculated by using a multiple –regression –based assignment approach, that it was possible to locate more than 70% of Italian individuals within 400 km of their reported area of origin and more than 90% within 800 km of their origin. Our PC analysis (Figure S2) seems to confirm these observations. It must also be emphasized that the results of this work were obtained using common SNPs and a more efficient geographic clustering could be reached using low –frequency alleles or haplotypes.

The overall F_st_ distribution fits with the PC analysis of the first two component. The F_st_ among the Italian macro-areas is moderate (F_st_≤0.001) when considering the Italian Peninsula, but is more pronounced between Sardinia and the other macro-areas (F_st_ = 0.004) (Table 2). This is in agreement with observations by other authors [15], [16] who reported that the average level of differentiation across Europe at each SNP is minimal (average F_st_ = 0.004 between different countries). A certain degree of genetic homogeneity shown by the F_st_ analysis, and by the partial overlapping of the distribution of the pairwise IBS within each of the Italian subpopulations, can be possibly explained by serial historical events and shared ancestry. The F_st_ values presented here are lower than those published by Nelis et al. 2009 using 270K SNPs; they reported that the Southern Italian population sample showed an F_st_ value of 0.005 compared to the Northern Italy sample. The highest value of this pairwise F_st_ matrix was found between the Finns from Kuusamo and Southern Italy (F_st_ = 0.023).

*ADMIXTURE* analysis confirms that there was no clear separation between Northern and Central Italy, at least as considered as macro-areas. Additional comparison of the distribution of pair-wise identity-by-state within each of the four populations and *ADMIXTURE* analysis clarified that this is not an artifact of the PC analysis. However, the PC and *ADMIXTURE* analysis results could be due to the sparse geographical coverage of our samples, especially for the Central and Northern macro-areas. In fact, many of the individuals (N = 413) in the North Italian sample analyzed in this study were from Piedmont- a North West Italian region- that has historically been affected by intense migration. At the same time, many individuals in the Central Italy macro-area (113 samples) are settled in Tuscany, an administrative region which is at the border with northern regions.

An intriguing result of the *ADMIXTURE* analysis was the proportion of ancestry in Sardinia, an ancestry shared with all the European and Northern African populations included in this analysis but with the highest level in Sardinia (Figure 3 crimson colour).

This average admixture proportion is widespread across all over the Sardinia island, with no geographic clustering, underlining an internal genetic homogeneity among the Sardinians. At the same time, this admixture proportion could be the signature of a common ancient genetic background of all the continental European populations but the isolation of the Sardinians have preserved this ancestry. The recent sequencing of the Iceman's genome, argues strongly in favor of the hypothesis that at least continental Europeans, living 5,300 years ago, were more similar to the current Sardinians [61].

The average admixture proportions for Northern European ancestry within current Sardinian population is 14.3% with some individuals exhibiting very low Northern European ancestry (less than 5% in 36 individuals on 268 accounting the 13% of the sample).

It is known that the major components are influenced by geographic clustering and secondly from areas with strong LD [17], and more precisely PC1 and PC2 are manly influenced by geography [15], [17]) and PC3 and PC4 indeed may be influenced by large scale genome structural variation, as the HLA region or 8p23 or 15q24 and 17q21.31 and many others. In order to verify which genomic region mainly contribute to each PC we plotted the contribution of each SNP to the first four PCs (eigenvalues) against the genomic location (Figure S4). The major contribution for PC4 was provided by 163 SNPs located on 8p23. The inversion of 4 Mb on chr 8 (8p23) is perhaps the largest inversion included in our genome. For this reason PC3 and PC4 do not display a geographical clustering, but seem to organize into three groups, also underlined by a K-means analysis (Figure S3 panel A and B). Once this evidence was obtained, we selected just these 163 markers for use in PCA1/PC2 and K-means analysis (Figure S5). We then estimated the percentage of inverted-homozygous or heterozygous or homozygous non-inverted to 8p23.1 using six samples previously typed by HapMap as a reference.

When the combined information across many loci and many individuals is used, for example in the λ**_GC_** analysis a higher degree of fine-scale population structure can be revealed. Systematic differences in sampling and genotyping are potential confounders, and may introduce a bias in association studies. The degree of genetic substructure between population pairs has been also measured by inflation factor (λ**_GC_**) statistics [52], [62]. For example, between Northern and Central Italy λ**_GC_** = 1.12, and in cases from Sardinia and controls from Northern Italy the genomic control inflation factor was 3.040 (Table 2).

Within Italy allele frequency differences warrant caution when matching controls and cases, especially when involving individuals with Sardinian descent.

In conclusion, autosomal GWAS data, confirm that the genetic structure of the Italian population was strongly influenced by of the geographical distance. Moreover all Italian subpopulations show inflation factors among the largest within Europe, second only to the Finns [20]. Our work also described the appealing potential of reconstructing the genetic structure of Italy by using existing collections of samples with genome-wide data, even when a reduced amount of information concerning the ancestral background of the sample donors is available. The geographical resolution presented in this study, which use a reduced sample size, also demonstrates the possibility of detecting subtle population structures using samples where only the birth place is know. Hence, further National and International collaborative initiatives should be developed in order to most effectively exploit existing genomic data. However, a higher level of resolution can only be achieved by increasing the sample size, including subjects with well-defined geographical origins and selected local surnames, and using SNP genotyping platforms containing low-frequency alleles, in order to have a more balanced and complete representation of the Italian regions.

## Supporting Information

Figure S1 **Top 100 PC and associated Eigenvalues in different datasets.**Top 100 PC and associated Eigenvalues for European dataset (a), Italian dataset (b) and Italian dataset without Sardinia (c).
(TIF)

Figure S2 **Hidden population structure within the Italian dataset.** Scatter plot of the first two eigenvectors based on 125,799 autosomal SNPs and 1,012 individuals. Colors represent the four different macro-areas; green- Southern Italy (Apulia, Calabria/Sicily, Campania, Basilicata), red- Central Italy (Tuscany, Lazio, Emilia Romagna and Abruzzo/Marche), black- Northern Italy (Piedmont,Liguria, Aosta Valley and Lombardy), blue- Sardinia (these samples were labeled for the linguistic area). Subjects are symbol- labeled by municipality. Information on municipality was not used for calculations.
(TIF)

Figure S3 **Italian population projected scatter plot of the PC3 and PC4. Panel A** Analysis based on 125,799 autosomal SNPs and 1,012 individuals. Color code shows different Italian subpopulations; green: Southern Italy, red: Central Italy, black: Northern Italy, blue: Sardinia.Panel B K-mean (K = 3) of PC3/PC4. Different colors shows the three diffent clusters.
(TIF)

Figure S4 **Variable contribution of each SNP to the first four PC against the genomic location.** Manhattan plot done in the Italian data set, the top SNPs in panel d localize to chromosome 8 inside the *8p23* region.
(TIF)

Figure S5 **Individuals predicted to be homozygous inverted or heterozygous or homozygous non-inverted for the 8p23.1.** Panel A Scatter plot of the PC1 and PC2 done using only SNPs located inside the *8p23* region (163 markers). Panel B Individuals predicted to be homozygous inverted or heterozygous or homozygous non-inverted using K mean clustering, K = 3. The frequency of homozygous inverted (light blue triangles) was of 21%, of homozygous non-inverted (orange triangles) was of 31.19%, heterozygous of 47.9%. Individuals from HapMap used to confirm the predictions for the 8p23.1 were respectively NA12815 (homozygous inverted); NA11992 and NA12057 (homozygous non-inverted); NA11993 NA06993 and NA11994 (heterozygous).
(TIF)

Figure S6 **Model-based ancestry analysis based on a subset from HGDP-CEPH and HapMap CEU data on 1260 individuals.** Ancestry for each individual was inferred with ADMIXTURE [50] from K = 2 to K = 9.
(TIF)

Figure S7 **Cross-validation error plot from the** ***ADMIXTURE*** **program.** Populations coming from the Italian dataset plus HGDP-CEPH (Palestinian; Druze; Mozambite; Bedouins; French) and some Hapmap populations. K = 1–10.
(TIF)

Table S1 **Number of SNPs inside each panel before and after a filter for SNP call rate.** SNPs intersection between the six studies was 163,355. After a filtering for minor allele frequency (MAF>0.01) the number of polymorphisms was reduced to 163,350. A subset of 163,095 SNPs passed *Hardy-Weinberg equilibrium*. A subsequent dataset of 125,799 SNPs after *Linkage Disequilibrium* pruning were used for PC analysis, F_st_, genomic control estimation and IBS analysis.
(DOC)

Table S2 **Correlation between PC's score and genetic/geographical values.**Correlation between PC1 score, PC2 score, PC3 score PC4 score and genetic distance (IBS) and latitude, longitude and geographical distance (great circle distance) within the Italian dataset. All the correlation values were significative (p-value less than 2.2 e^−16^).
(DOC)
