# Supplementary figures and images for: An Overview of the Genetic Structure within the Italian Population from Genome-Wide Data
Source: PLoS One. 2012 Sep 12;7(9):e43759. doi: 10.1371/journal.pone.0043759 (PMC3440425; doi:10.1371/journal.pone.0043759)

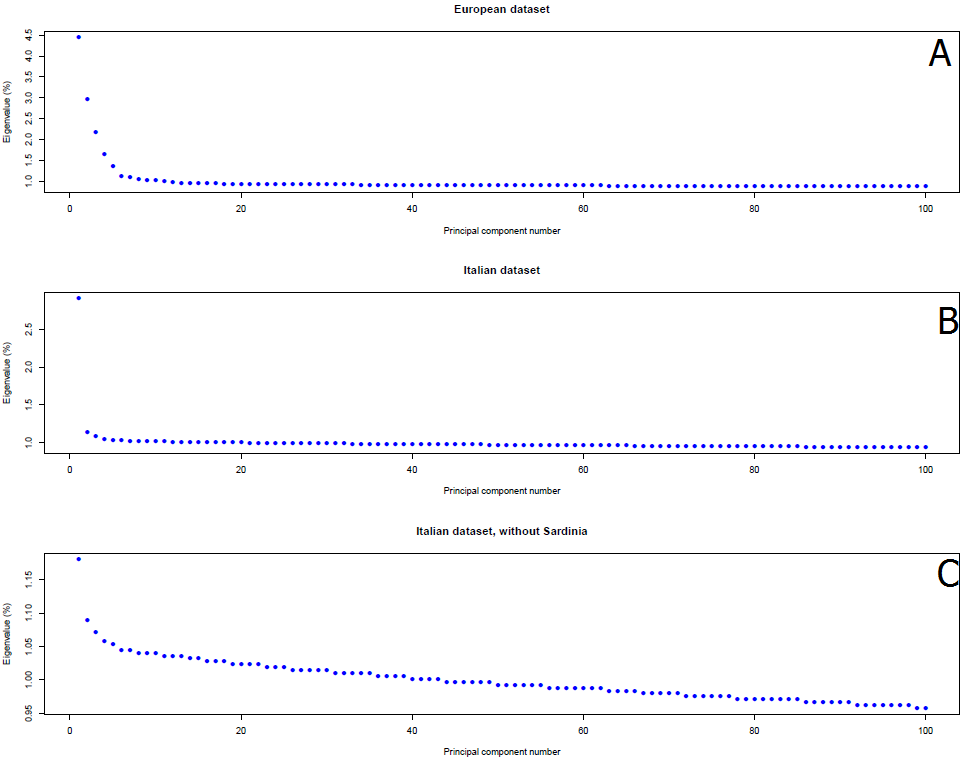

Supplement: Figure S1 — Top 100 PC and associated Eigenvalues in different datasets.Top 100 PC and associated Eigenvalues for European dataset (a), Italian dataset (b) and Italian dataset without Sardinia (c). (TIF) [file pone.0043759.s001.tif]

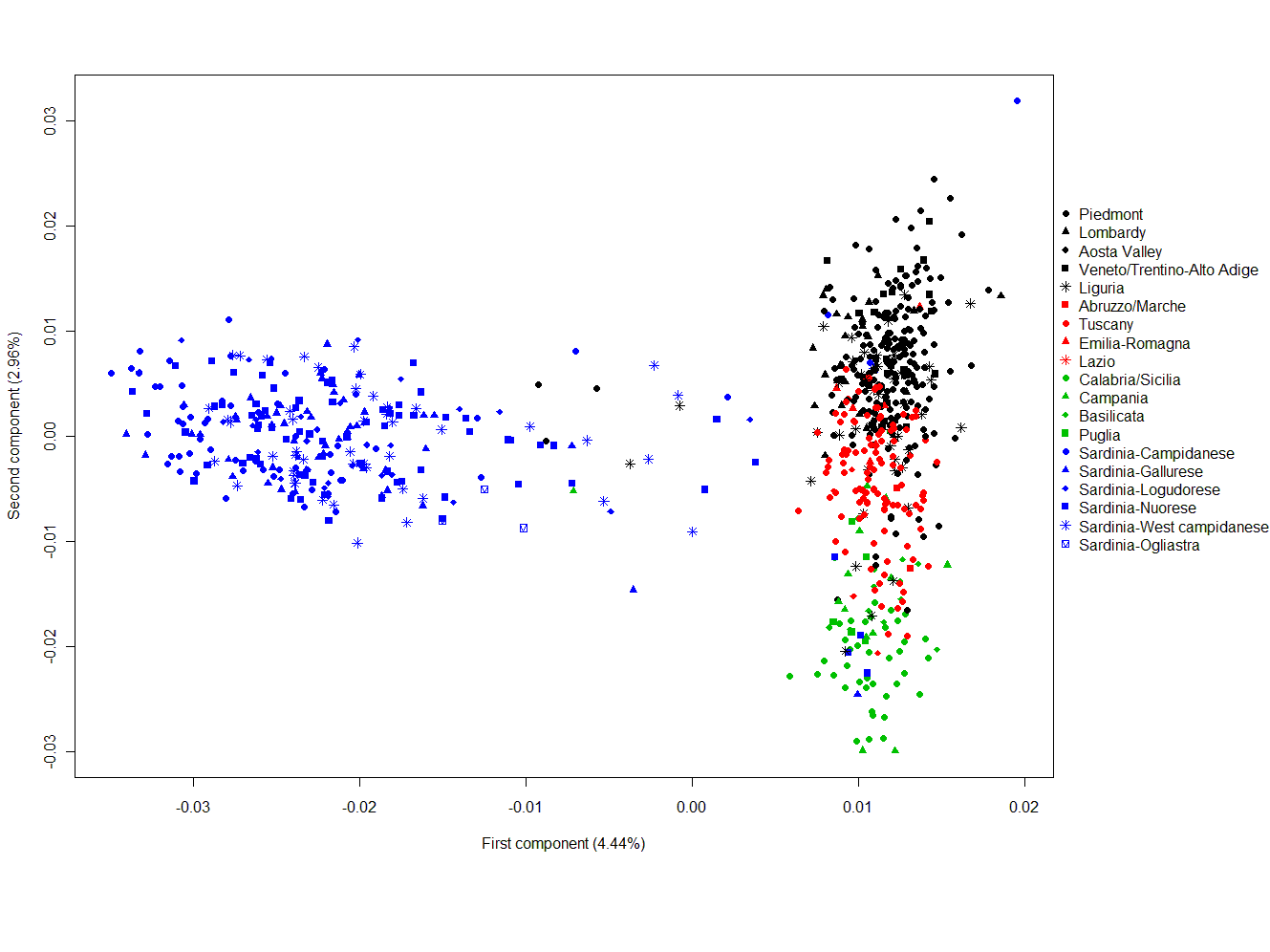

Supplement: Figure S2 — Hidden population structure within the Italian dataset. Scatter plot of the first two eigenvectors based on 125,799 autosomal SNPs and 1,012 individuals. Colors represent the four different macro-areas; green- Southern Italy (Apulia, Calabria/Sicily, Campania, Basilicata), red- Central Italy (Tuscany, Lazio, Emilia Romagna and Abruzzo/Marche), black- Northern Italy (Piedmont,Liguria, Aosta Valley and Lombardy), blue- Sardinia (these samples were labeled for the linguistic area). Subjects are symbol- labeled by municipality. Information on municipality was not used for calculations. (TIF) [file pone.0043759.s002.tif]

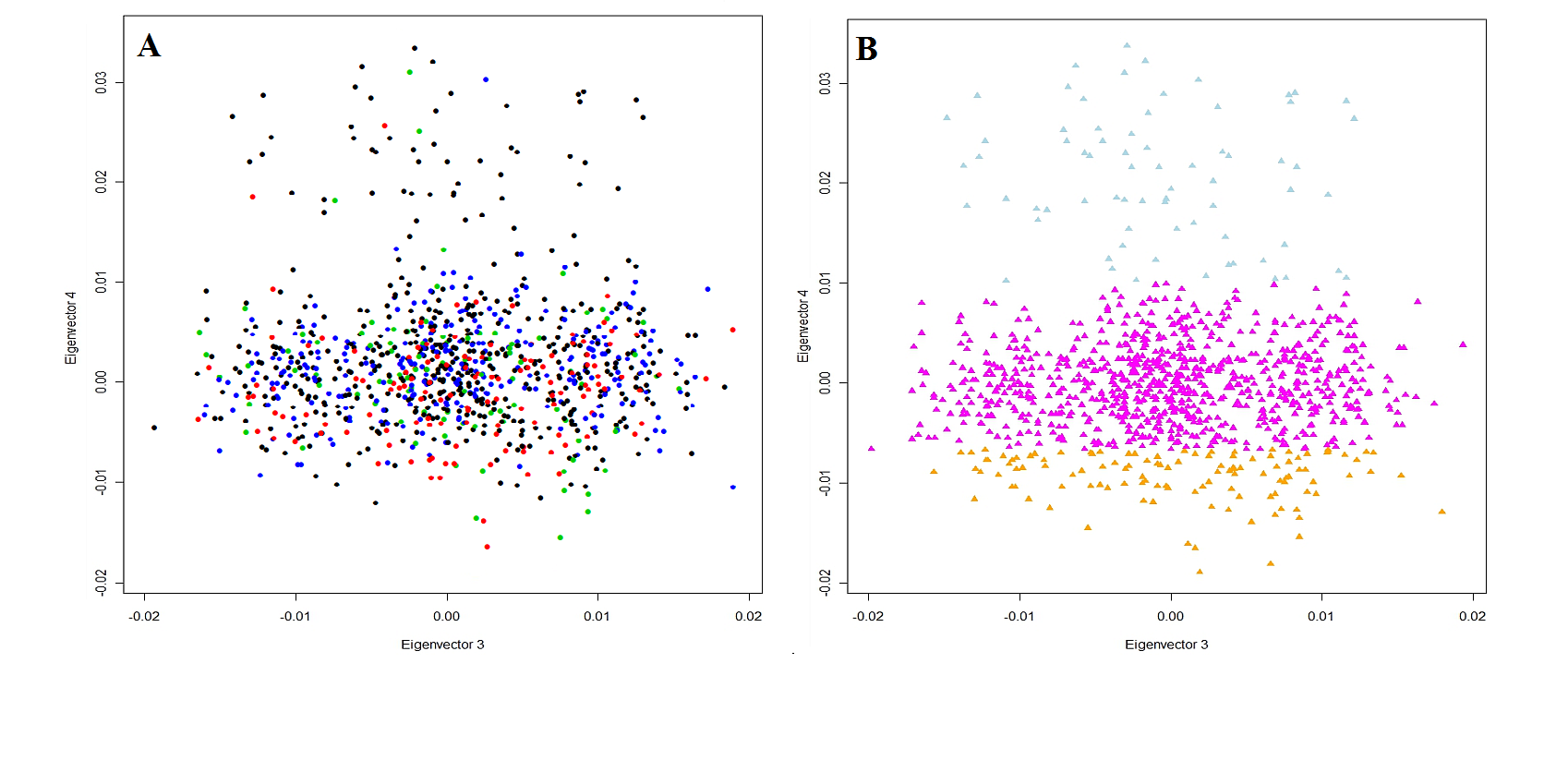

Supplement: Figure S3 — Italian population projected scatter plot of the PC3 and PC4. Panel A Analysis based on 125,799 autosomal SNPs and 1,012 individuals. Color code shows different Italian subpopulations; green: Southern Italy, red: Central Italy, black: Northern Italy, blue: Sardinia.Panel B K-mean (K = 3) of PC3/PC4. Different colors shows the three diffent clusters. (TIF) [file pone.0043759.s003.tif]

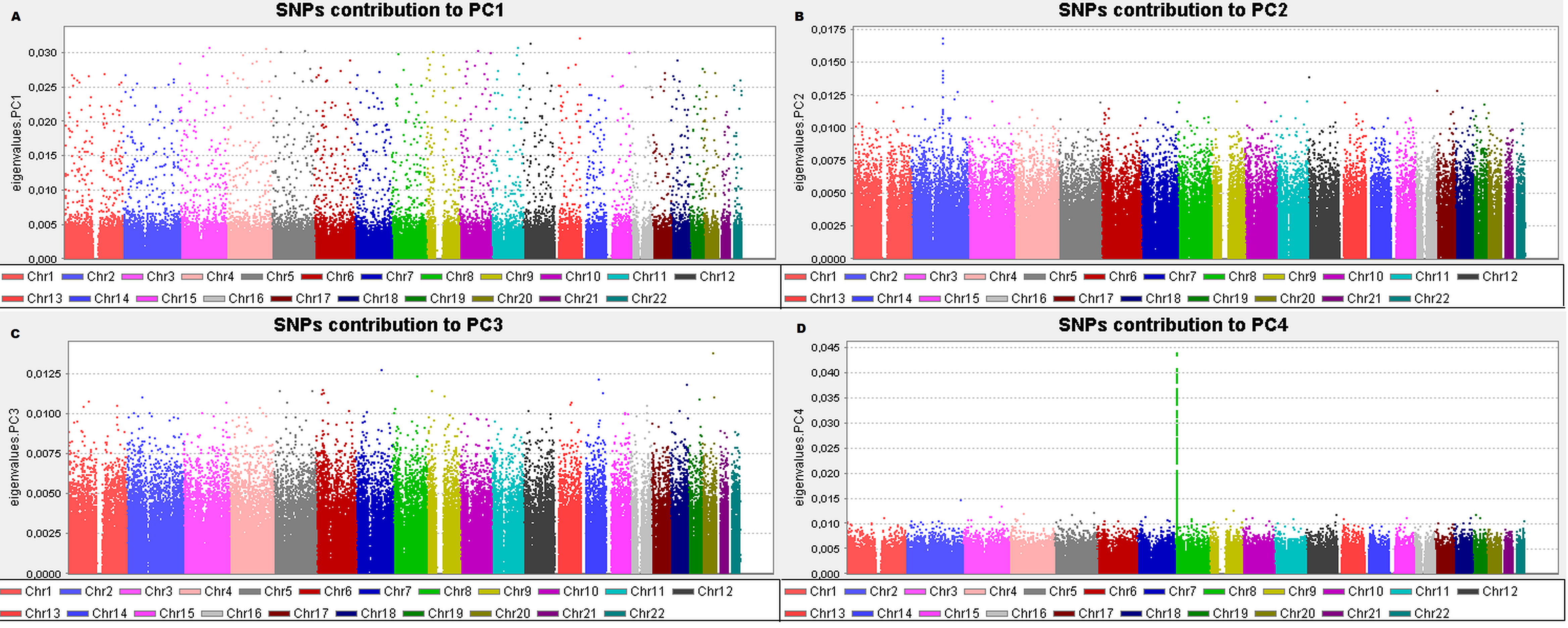

Supplement: Figure S4 — Variable contribution of each SNP to the first four PC against the genomic location. Manhattan plot done in the Italian data set, the top SNPs in panel d localize to chromosome 8 inside the 8p23 region. (TIF) [file pone.0043759.s004.tif]

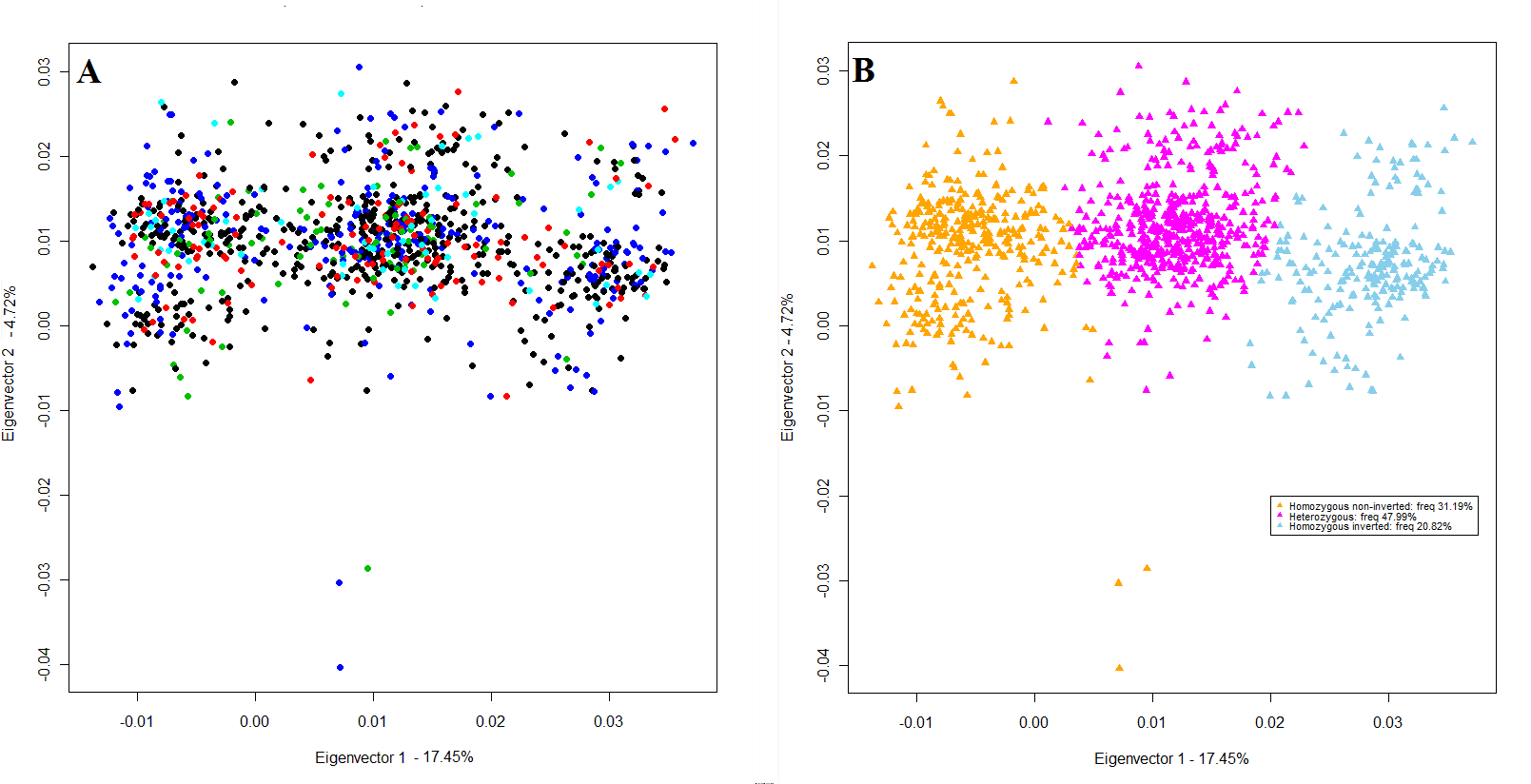

Supplement: Figure S5 — Individuals predicted to be homozygous inverted or heterozygous or homozygous non-inverted for the 8p23.1. Panel A Scatter plot of the PC1 and PC2 done using only SNPs located inside the 8p23 region (163 markers). Panel B Individuals predicted to be homozygous inverted or heterozygous or homozygous non-inverted using K mean clustering, K = 3. The frequency of homozygous inverted (light blue triangles) was of 21%, of homozygous non-inverted (orange triangles) was of 31.19%, heterozygous of 47.9%. Individuals from HapMap used to confirm the predictions for the 8p23.1 were respectively NA12815 (homozygous inverted); NA11992 and NA12057 (homozygous non-inverted); NA11993 NA06993 and NA11994 (heterozygous). (TIF) [file pone.0043759.s005.tif]

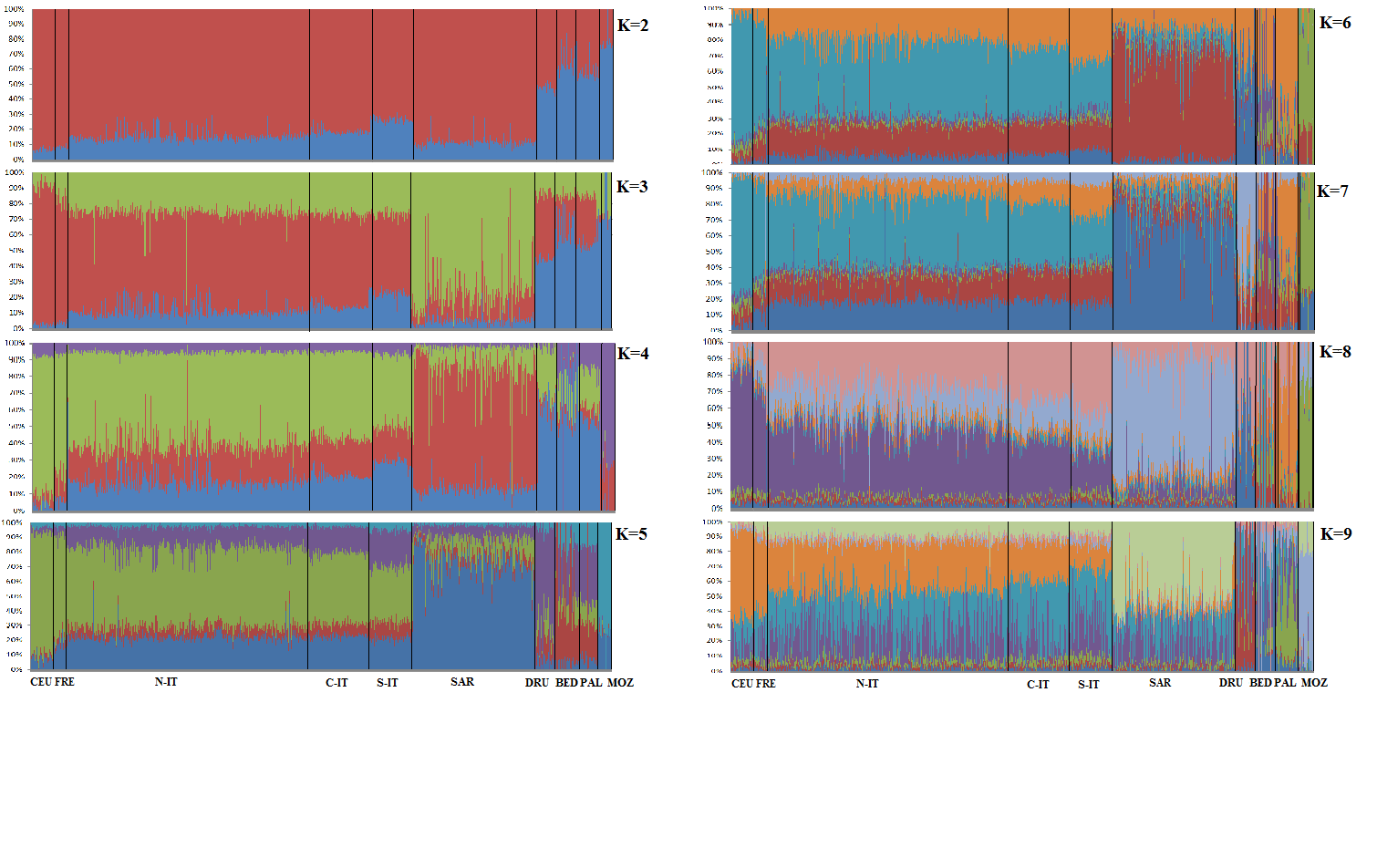

Supplement: Figure S6 — Model-based ancestry analysis based on a subset from HGDP-CEPH and HapMap CEU data on 1260 individuals. Ancestry for each individual was inferred with ADMIXTURE [50] from K = 2 to K = 9. (TIF) [file pone.0043759.s006.tif]

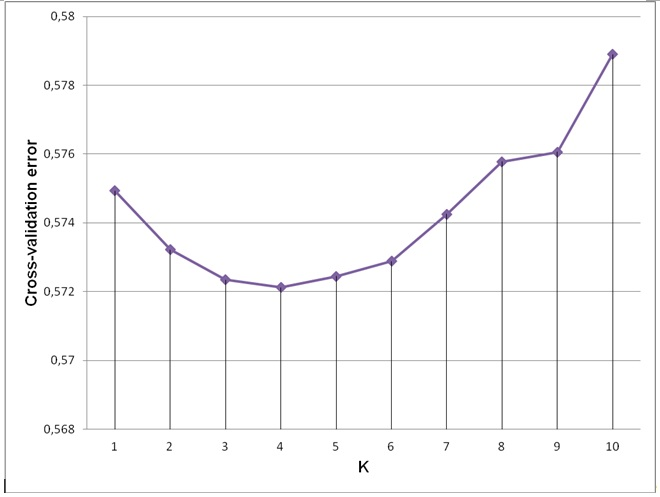

Supplement: Figure S7 — Cross-validation error plot from the ADMIXTURE program. Populations coming from the Italian dataset plus HGDP-CEPH (Palestinian; Druze; Mozambite; Bedouins; French) and some Hapmap populations. K = 1–10. (TIF) [file pone.0043759.s007.tif]
