# Supplementary material for: An Overview of the Genetic Structure within the Italian Population from Genome-Wide Data
Source: PLoS One. 2012 Sep 12;7(9):e43759. doi: 10.1371/journal.pone.0043759 (PMC3440425; doi:10.1371/journal.pone.0043759)
Supplement: Table S1 — Number of SNPs inside each panel before and after a filter for SNP call rate. SNPs intersection between the six studies was 163,355. After a filtering for minor allele frequency (MAF>0.01) the number of polymorphisms was reduced to 163,350. A subset of 163,095 SNPs passed Hardy-Weinberg equilibrium. A subsequent dataset of 125,799 SNPs after Linkage Disequilibrium pruning were used for PC analysis, Fst, genomic control estimation and IBS analysis. (DOC) [file pone.0043759.s008.doc]

|  | Initial number of SNPs | After a filter for SNP call rate > 95% |
| --- | --- | --- |
| HapMap | 1440616 | 1440616 |
| HGDP | 660918 | 659539 |
| MESO | 330879 | 330531 |
| Our sample | 1140419 | 1001060 |
| GEO-IT | 657366 | 556276 |
| HYPERGENES | 1198070 | 1135472 |
