# Supplementary material for: An Overview of the Genetic Structure within the Italian Population from Genome-Wide Data
Source: PLoS One. 2012 Sep 12;7(9):e43759. doi: 10.1371/journal.pone.0043759 (PMC3440425; doi:10.1371/journal.pone.0043759)
Supplement: Table S2 — Correlation between PC's score and genetic/geographical values.Correlation between PC1 score, PC2 score, PC3 score PC4 score and genetic distance (IBS) and latitude, longitude and geographical distance (great circle distance) within the Italian dataset. All the correlation values were significative (p-value less than 2.2 e−16). (DOC) [file pone.0043759.s009.doc]

|  | PC1 score | PC2 score | PC3 score | PC4 score | IBS distance |
| --- | --- | --- | --- | --- | --- |
| Latitude | 0.422 | 0.320 | 0.007 | 0.079 | 0.307 |
| Longitude | -0.063 | 0.496 | 0.028 | 0.163 | 0.320 |
| Geographical distance | 0.319 | 0.490 | 0.024 | 0.128 | 0.408 |
